# Supplementary material for: Insulin-like Growth Factor-1 and IGF Binding Proteins Predict All-Cause Mortality and Morbidity in Older Adults
Source: Cells. 2020 Jun 1;9(6):1368. doi: 10.3390/cells9061368 (PMC7349399; doi:10.3390/cells9061368)
Supplement: Supplementary file 1 [file cells-09-01368-s001.pdf]

Supplemental Material

Table S1. Baseline prevalence of age-related morbidities.

	All	Male	Female
Cardiovascular disease, n (%)	107 (12.7)	88 (23.0)	19 (4.1)
Diabetes, n (%)	88 (10.5)	53 (13.9)	35 (7.6)
Cancer, n (%)	186 (22.1)	80 (20.9)	106 (23.1)
MDCI, n (%)	25 (3.0)	12 (3.1)	13 (2.8)

MDCI, multiple-domain cognitive impairment.

Table S2. Baseline Characteristics of High and Low IGF-1 Groups. All p-values are for comparisons between the low IGF-1 and high IGF-1 groups.

	Combined	Low IGF-1	High IGF-1	p-value
**All**				
**Number of Individuals, n (%)**	761	369 (48.5)	392 (51.5)	0.43
**Age (years), mean ± SD**	76.1 ± 6.8	76.6 ± 6.9	75.6 ± 6.7	0.04
**IGF-1 (ng/mL), mean ± SD**	117 ± 38	88 ± 20	145 ± 30	< 0.001
**Deaths, n (%)**	106 (13.9)	53 (14.4)	53 (13.5)	0.74
	**Males**			
**Number of Individuals, n (%)**	345	165 (47.8)	180 (52.2)	0.45
**Age (years), mean ± SD**	76.2 ± 7.0	77.2 ± 7.0	75.4 ± 6.9	0.02
**IGF-1 (ng/mL), mean ± SD**	127 ± 39	96 ± 19	156 ± 29	< 0.001
**Deaths, n (%)**	57 (16.5)	32 (19.4)	25 (13.9)	0.17
	**Females**			
**Number of Individuals, n (%)**	416	204 (49.0)	212 (51.0)	0.73
**Age (years), mean ± SD**	76.0 ± 6.7	76.2 ± 6.8	75.8 ± 6.5	0.57
**IGF-1 (ng/mL), mean ± SD**	108 ± 36	80.5 ± 19	135 ± 28	< 0.001
**Deaths, n (%)**	49 (11.8)	21 (10.3)	28 (13.2)	0.36

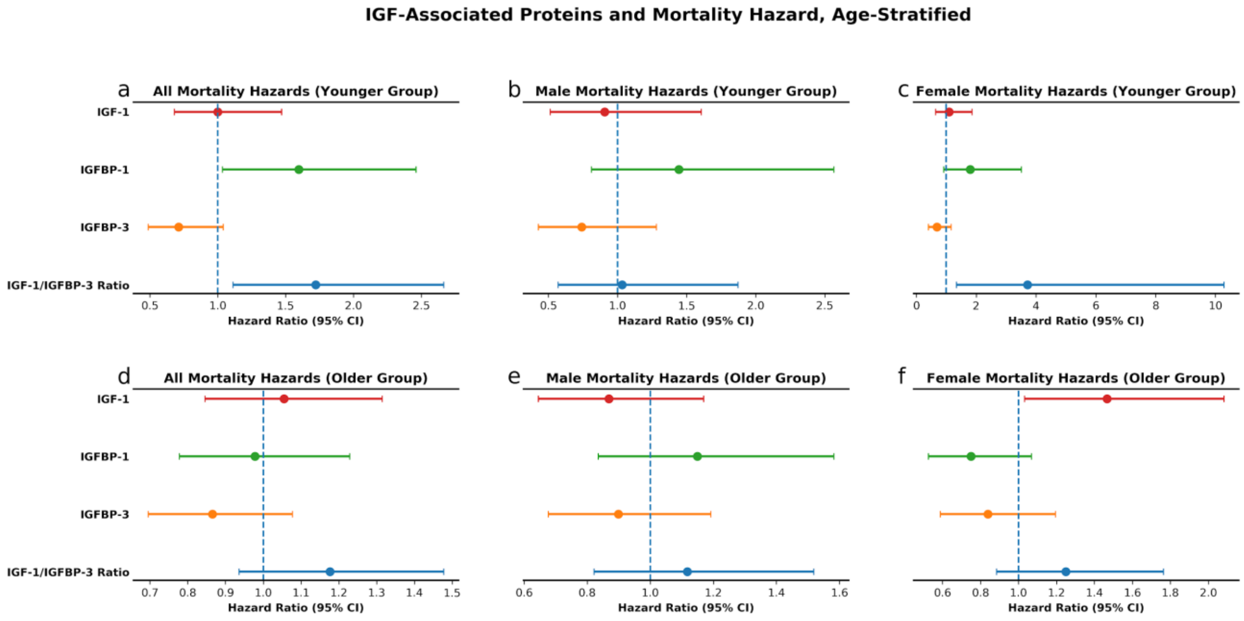

Figure S1. IGF-Associated Proteins and Mortality Hazard, Age-Stratified. Incident mortality hazards for combined cohort, adjusted for age and sex (**a**), males, adjusted for age (**b**), and females, adjusted for age (**c**) in individuals younger than age 75.51 years at baseline (Younger group). Incident

mortality hazard for combined cohort, adjusted for age and sex (**d**), males, adjusted for age (**e**), and females, adjusted for age (**f**) in individuals older than 75.51 years at baseline (Older group). All analyses are for high levels of IGF-associated proteins as compared to individuals with low levels (biochemical measurements dichotomized by sex-specific medians).

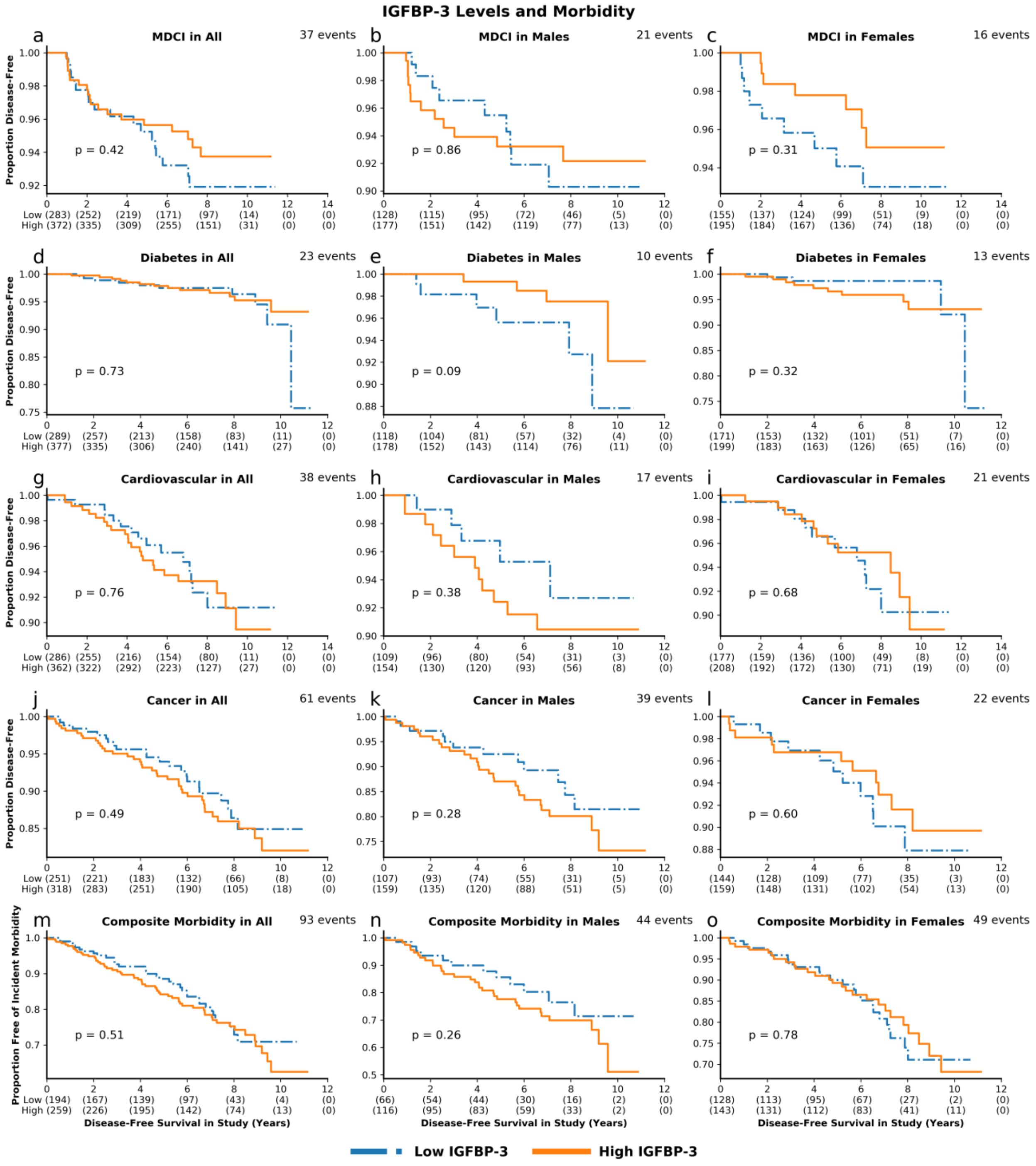

Figure S2. IGFBP-3 Levels and Morbidity. Unadjusted survival curves for MDCI (**a**. combined cohort; **b**. males; **c**. females), diabetes (**d**–**f**), cardiovascular disease (**g**–**i**), cancer (**j**–**l**), and composite incident morbidity (m-o) in individuals with high and low levels of IGFBP-3.

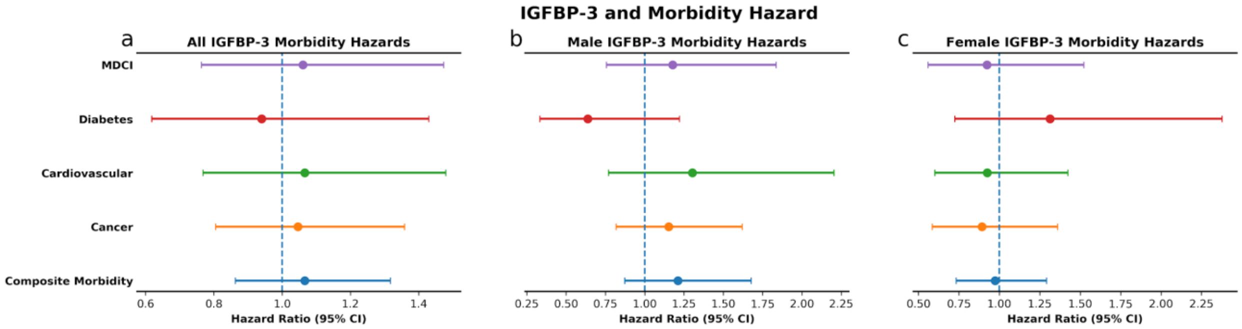

Figure S3. IGFBP-3 and Morbidity Hazard. Sex and age-adjusted incident morbidity hazards for all individuals (**a**) and age-adjusted survival hazard for males (**b**) and females (**c**) with high levels of IGFBP-3 as compared to individuals with low levels.

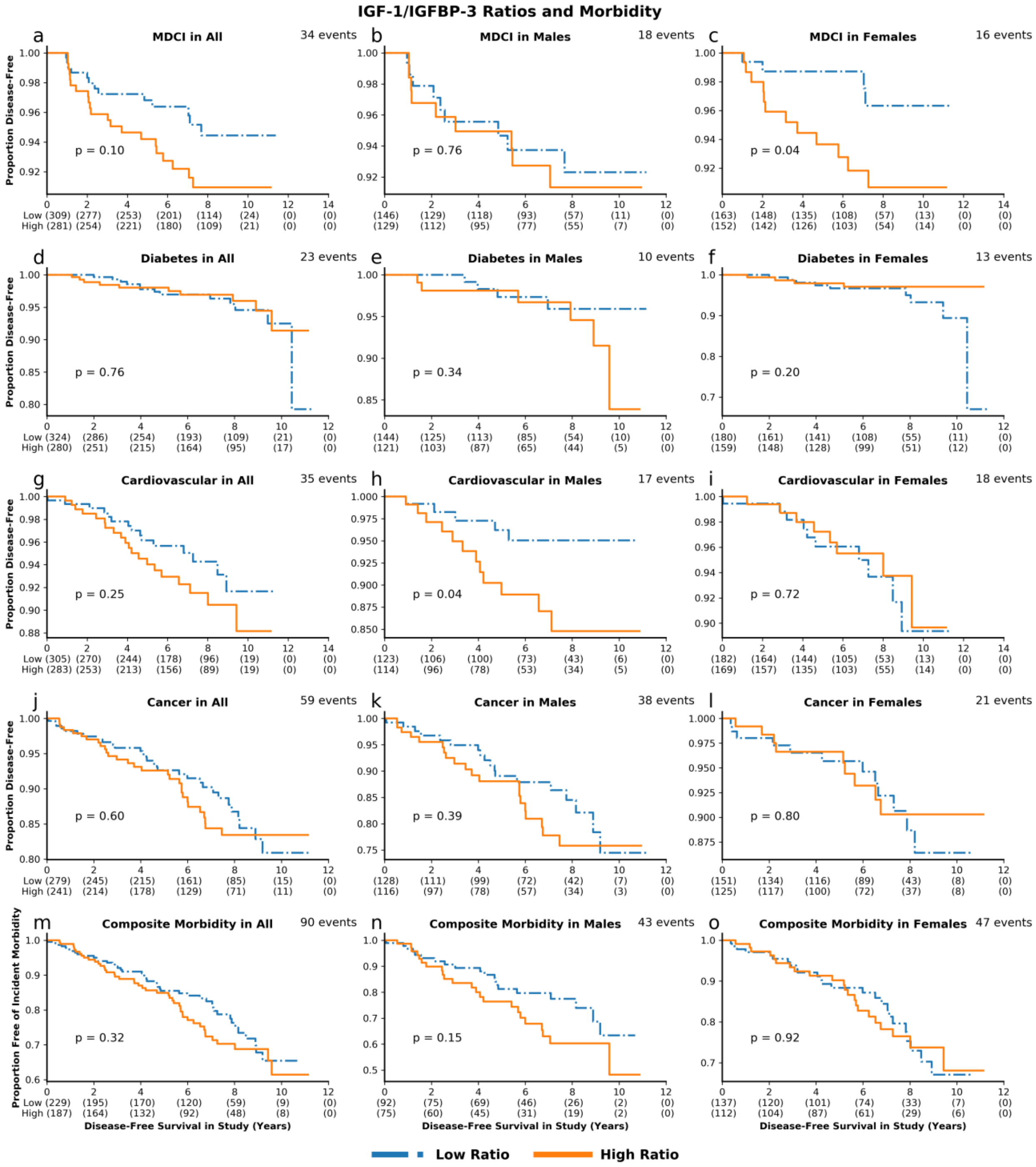

Figure S4. IGF-1/IGFBP-3 Molar Ratios and Morbidity. Unadjusted survival curves for MDCI (**a**. combined cohort; **b**. males; **c**. females), diabetes (**d**-**f**), cardiovascular disease (**g**-**i**), cancer (**j**-**l**), and composite incident morbidity (**m**-**o**) in individuals with high and low IGF-1/IGFBP-3 molar ratios.

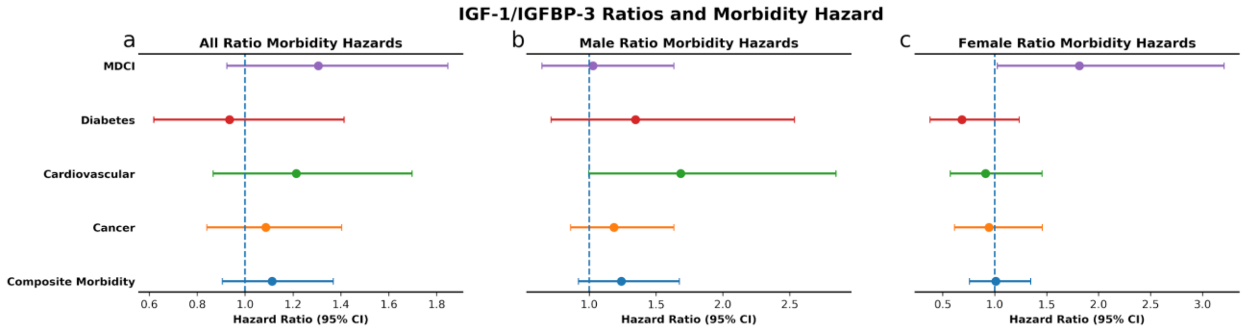

Figure S5. IGF-1/IGFBP-3 Molar Ratios and Morbidity Hazard**.** Sex and age-adjusted incident morbidity hazards for all individuals (**a**) and age-adjusted survival hazard for males (**b**) and females (**c**) with high molar ratios of IGF-1/IGFBP-3 as compared to individuals with low molar ratios.
